# Therapeutic Effects of Cornuside on Particulate Matter–Induced Lung Injury

**DOI:** 10.3390/ijms24054979

**Published:** 2023-03-04

**Authors:** Go Oun Kim, Eui Kyun Park, Dong Ho Park, Gyu Yong Song, Jong-Sup Bae

**Affiliations:** 1 College of Pharmacy, Research Institute of Pharmaceutical Sciences, Kyungpook National University, Daegu 41566, Republic of Korea; 2 Department of Pathology and Regenerative Medicine, School of Dentistry, Kyungpook National University, Daegu 41940, Republic of Korea; 3 Department of Ophthalmology, School of Medicine, Kyungpook National University, Kyungpook National University Hospital, Daegu 41944, Republic of Korea; 4 College of Pharmacy, Chungnam National University, 99 Daehak-ro, Yuseong-gu, Daejon 34134, Republic of Korea

**Keywords:** cornuside, particulate matter, lung injury, TLR4–mTOR–autophagy pathway

## Abstract

Particulate matter (PM) is a mixture comprising both organic and inorganic particles, both of which are hazardous to health. The inhalation of airborne PM with a diameter of ≤2.5 μm (PM_2.5_) can cause considerable lung damage. Cornuside (CN), a natural bisiridoid glucoside derived from the fruit of *Cornus officinalis* Sieb, exerts protective properties against tissue damage via controlling the immunological response and reducing inflammation. However, information regarding the therapeutic potential of CN in patients with PM_2.5_-induced lung injury is limited. Thus, herein, we examined the protective properties of CN against PM_2.5_-induced lung damage. Mice were categorized into eight groups (n = 10): a mock control group, a CN control group (0.8 mg/kg mouse body weight), four PM_2.5_+CN groups (0.2, 0.4, 0.6, and 0.8 mg/kg mouse body weight), and a PM_2.5_+CN group (0.2, 0.4, 0.6, and 0.8 mg/kg mouse body weight). The mice were administered with CN 30 min following intratracheal tail vein injection of PM_2.5_. In mice exposed to PM_2.5_, different parameters including changes in lung tissue wet/dry (W/D) lung weight ratio, total protein/total cell ratio, lymphocyte counts, inflammatory cytokine levels in the bronchoalveolar lavage fluid (BALF), vascular permeability, and histology were examined. Our findings revealed that CN reduced lung damage, the W/D weight ratio, and hyperpermeability caused by PM_2.5_. Moreover, CN reduced the plasma levels of inflammatory cytokines produced because of PM_2.5_ exposure, such as tumor necrosis factor (TNF)-α, interleukin (IL)-1β, and nitric oxide, as well as the total protein concentration in the BALF, and successfully attenuated PM_2.5_-associated lymphocytosis. In addition, CN substantially reduced the expression levels of Toll-like receptors 4 (TLR4), MyD88, and autophagy-related proteins LC3 II and Beclin 1, and increased protein phosphorylation of the mammalian target of rapamycin (mTOR). Thus, the anti-inflammatory property of CN renders it a potential therapeutic agent for treating PM_2.5_-induced lung injury by controlling the TLR4–MyD88 and mTOR–autophagy pathways.

## 1. Introduction

Due to industry emissions, the amount of global anthropogenic-driven air pollution has increased recently [1]. Suspended particulate matter (PM) with a diameter of ≤2.5 μm (PM_2.5_) can cause injuries to the respiratory and circulatory systems, and it is a well-known indicator of air pollution. Due to its small size, 96% of PM_2.5_ can be retained in the lungs for decades [2]. Heavy metals, polycyclic aromatic hydrocarbons, oxygenated volatile organic compounds, etc. contribute to the harmful effects of PM_2.5_ [3]. Respiratory diseases, including asthma, acute lung damage, and chronic obstructive pulmonary disease, have been associated with PM_2.5_-induced inflammation. Additionally, the release of various cytokines and chemokines, including interleukins (ILs) and tumor necrosis factor (TNF)-α, is mediated by inflammation caused by PM_2.5_ [3,4,5]. Thus, there is an urgent need to develop novel preventive and treatment strategies for respiratory diseases, especially owing to the strong association between PM_2.5_ exposure and the risk of asthma and the incidence and mortality of lung cancer [6].

Lung damage involves a complex cascade of molecular processes, including the activation of the Toll-like receptor 4 (TLR4), a key regulator capable of triggering innate immune responses and controlling inflammatory mediators [7]. Reportedly, PM can increase inflammatory mediator production via TLR4 pathway activation [8]. PM inhibits the mammalian target of rapamycin (mTOR), a sensor of cellular nutritional status, and increases cellular oxidative stress, thereby leading to cellular apoptosis and autophagy and destroying cellular components [9]. Autophagy is an essential lysosome-dependent mechanism involved in cell homeostasis, which is reportedly associated with protein aggregation, organelle damage, and intracellular pathogen turnover [10]. In addition, autophagy has been linked to lung disorders and, therefore, is crucial for controlling pulmonary diseases [11]. Thus, TLR4 and autophagy suppression may be therapeutically beneficial for pulmonary damage.

Traditional eastern medicine has extensively employed cornuside (CN), a bisiridoid glucoside derived from the plant *Cornus officinalis* Sieb. et Zucc., to treat inflammatory disorders and improve hemokinesis. Several pharmacological effects have been attributed to fruit extracts derived from *C. officinalis*, including anticancer, anti-inflammatory, and hepatoprotective properties [12,13]. For instance, CN prevents oxygen and glucose deprivation in cultured rat cortical cells and reduces the generation of proinflammatory and cell adhesion molecules in response to cytokines in human endothelial cells [12,13]. However, investigations into the mechanisms underlying the effects of CN on lung damage, histology, inflammation, and TLR4–autophagy pathways following PM_2.5_ exposure remain lacking. To fill this knowledge gap, we used a PM_2.5_ exposure mouse model to test our hypothesis that CN inhibits PM_2.5_-induced proinflammatory responses and autophagy in lung tissue cells and heals lung tissue damage by inhibiting the TLR4 and autophagy pathways.

## 2. Results

### 2.1. Effects of CN Lung Damage by PM_2.5_

**Figure 1:**
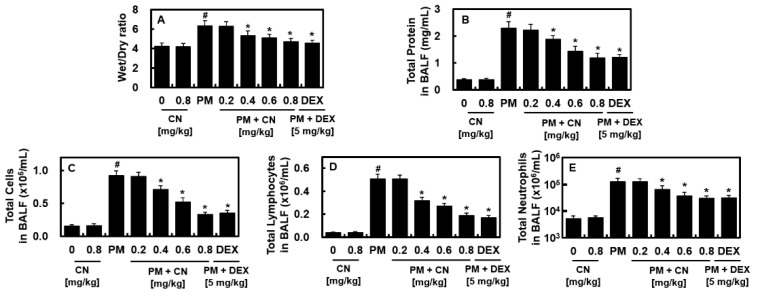
Effects of CN on PM_2.5_-induced lung damage. CN and dexamethasone (DEX) groups received intravenous injections 30 min after intratracheal PM_2.5_ instillation (10 mg/kg in 50 μL saline). The mice were euthanized 24 h following the PM_2.5_ injection, and their lung tissues and BALF were collected. The effects of different concentrations of CN and DEX were evaluated for the (**A**) W/D ratio, (**B**) total number of cells in the BALF, (**C**) total protein levels in the BALF, (**D**) lymphocyte, and neutrophils (**E**) count. Data were analyzed using ANOVA followed by Tukey’s post hoc test. Data are the means ± SD of three independent experiments (n = 3). ^#^ *p* < 0.01 versus control group and * *p* < 0.01 versus PM_2.5_ group.

The lung W/D weight ratio was calculated to determine the effects of CN on PM_2.5_-induced lung damage. This ratio increased in the PM_2.5_ group (Figure 1A) and decreased with the application of CN or dexamethasone (DEX, 5 mg/kg). The total protein levels in the BALF and inflammatory cell infiltration due to PM_2.5_ were also assessed. The PM_2.5_ group exhibited higher total protein levels and total cell, lymphocyte, and neutrophil counts in the mouse BALF than the control group (Figure 1B–E). However, after administering PM_2.5_ via endotracheal instillation, CN or DEX (5 mg/kg) therapy decreased the total cell and lymphocyte counts in the BALF. Similarly, the total protein levels in the BALF were decreased due to CN or DEX administration in a dose-dependent manner (Figure 1B).

**Figure 2:**
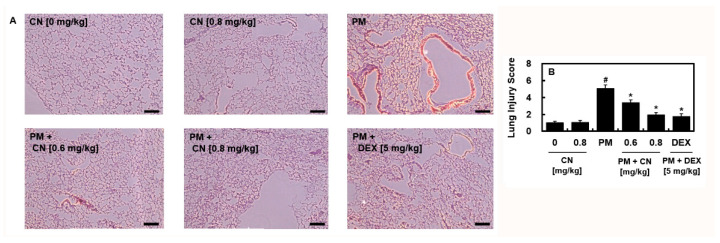
Effects of CN on PM_2.5_-induced lung injury. CN and DEX groups received intravenous injections 30 min after intratracheal PM_2.5_ instillation (10 mg/kg in 50 μL saline). The mice were euthanized 24 h following the PM_2.5_ injection, and their lung tissues and BALF were collected. (**A**) H&E staining was used to analyze histological changes in the lung tissue. Five representative photographs from each group were analyzed. Scale bar: 200 μm. Lung injury score (**B**). Data were analyzed using ANOVA followed by Tukey’s post hoc test. Data are the means ± SD of three independent experiments (n = 3). ^#^ *p* < 0.01 versus control group and * *p* < 0.01 versus PM_2.5_ group.

Changes in lung histology were examined using hematoxylin and eosin (H&E) staining to determine the protective effects of CN against PM_2.5_-induced lung damage. The alveolar wall was invaded and coated with the inflammatory cells from the PM_2.5_ group (Figure 2A). A lung injury score was calculated following treatment with various doses of CN or DEX (Figure 2B), and the results revealed that CN reduced inflammatory cell infiltration and protected the lungs from PM_2.5_-induced lung damage.

### 2.2. Effects of CN on Vascular Barrier Disruptive Responses by PM_2.5_

**Figure 3:**
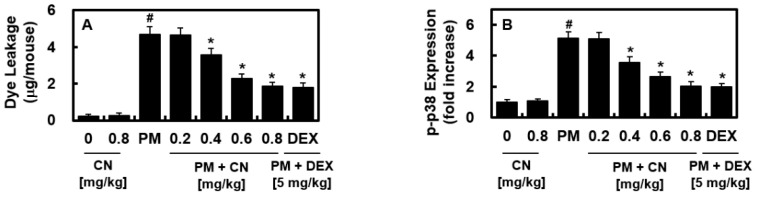
Effects of CN on PM_2.5_-induced vascular barrier disruptive response and p38 MAPK activation. CN and DEX groups received intravenous injections 30 min after intratracheal PM_2.5_ instillation (10 mg/kg in 50 μL saline). (**A**) Evans blue flux in the BALF (reported as g/mouse, n = 5) and (**B**) phosphorylated p38 (p-p38) levels in MLMVECs of mice were assessed via ELISA and used to determine the effects of CN or DEX on vascular hyperpermeability by PM_2.5_. Data were analyzed using ANOVA followed by Tukey’s post hoc test. Data are the means ± SD of three independent experiments (n = 3). ^#^ *p* < 0.01 versus control group and * *p* < 0.01 versus PM_2.5_ group.

The integrity of the vascular barrier can be compromised by PM [14], so this value was calculated to determine how CN may affect vascular disruptive reactions caused by PM_2.5_. Figure 3A demonstrates that dye leakage in the BALF increased after PM_2.5_ treatment and was reduced by CN or DEX (Figure 3A). We also observed that PM_2.5_ induced the phosphorylation of p38 MAPK, whereas CN or DEX treatment prevented it. The vascular damage response to inflammatory proteins is mediated by the p38 MAPK signaling pathway (Figure 3B).

### 2.3. Effects of CN on Pulmonary Inflammatory Responses by PM_2.5_

**Figure 4:**
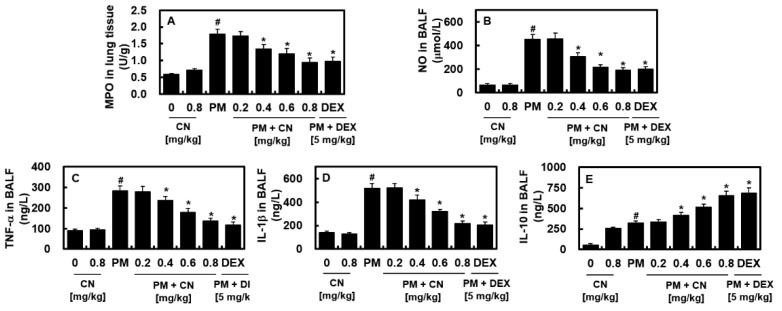
Effects of CN on PM_2.5_-induced pulmonary inflammation. CN and DEX groups received intravenous injections 30 min after intratracheal PM_2.5_ instillation (10 mg/kg in 50 μL saline). After the mice were killed 24 h following PM_2.5_ injection, their lung tissues and BALF were collected. Measurements include (**A**) MPO in lung tissue and (**B**) NO, (**C**) TNF-α, (**D**) IL-1β, and (**E**) IL-10 in the BALF. Data were analyzed using ANOVA followed by Tukey’s post hoc test. Data are the means ± SD of three independent experiments (n = 3). ^#^ *p* < 0.01 versus control group and * *p* < 0.01 versus PM_2.5_ group.

We examined the effects of CN against PM_2.5_-induced pulmonary inflammatory responses and observed that CN decreased PM_2.5_-induced vascular barrier breakdown in vivo (Figure 3). Increased lung MPO activity denotes neutrophil tissue infiltration, and inflammatory cytokines, such as NO, IL-1β, and TNF-α, are good indicators of inflammatory processes. Figure 4 shows that PM_2.5_ increased lung tissue MPO activity and NO, TNF-α, and IL-1β production in the BALF compared with the control group, which was reduced via CN or DEX therapy. To further confirm the anti-inflammatory property of CN, the levels of an anti-inflammatory cytokine (IL-10) were measured. Interestingly, we found that PM_2.5_ as well as CN alone led to increased levels of IL-10 in the BALF, and, after treatment with CN and PM_2.5_, there was a further increase in the levels of IL-10 as compared with control (Figure 4E). These results suggested that CN attenuated PM_2.5_-induced ALI by inhibiting inflammatory cytokines and up-regulating anti-inflammatory cytokines.

### 2.4. Effects of CN on Signaling Pathways by PM_2.5_

**Figure 5:**
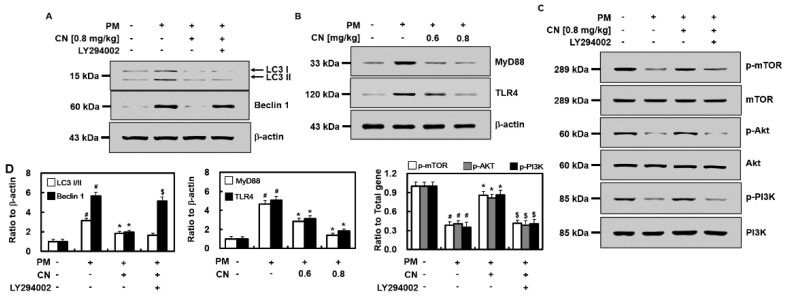
Effects of CN on PM_2.5_-induced signaling pathways. CN groups received an intravenous injection 30 min after intratracheal PM_2.5_ instillation_5_ (10 mg/kg in 50 μL saline). The mice were killed 24 h following the PM_2.5_ injection, and their lung tissues were collected. The expression levels of (**A**) LC3 and Beclin 1, (**B**) TLR4 and MyD88, and (**C**) p-mTOR, mTOR, p-Akt, Akt, pPI3K, and PI3K as shown via western blot analysis. Representative images from each group are shown (*n* = 3). (**D**) Densitometric intensities of each signaling pathway component normalized to β-actin or total protein. ^#^ *p* < 0.01 versus control group, * *p* < 0.01 versus PM_2.5_ group, and ^$^ *p* < 0.01 versus PM_2.5_ with CN group.

Western blot analysis was performed to examine the regulatory effects of CN on LC3 and Beclin 1. The PM_2.5_ group exhibited higher levels of LC3 II and Beclin 1 than the control group (Figure 5A,D). CN reduced the PM_2.5_-induced increase in LC3 and Beclin 1 expression levels in mouse lung tissue, suggesting that it can prevent autophagy caused by PM_2.5_. However, the LY294002 treatment partially reversed some of these effects. The lung tissues of the groups were assessed using western blotting analysis for TLR4, MyD88, p-mTOR, total mTOR, p-Akt, Akt, p-PI3K, and PI3K to understand the associations between the TLR4 and mTOR–autophagy pathways and the anti-inflammatory/antiautophagy effects of CN. PM_2.5_ intratracheal instillation increased TLR4 and MyD88 expression levels in the lung tissue (Figure 5B,D), which were decreased via CN therapy (0.8 mg/kg). The PM_2.5_ group exhibited lower levels of p-mTOR, p-Akt, and p-PI3K compared with the control group (Figure 5C,D). The present study suggests that CN can activate the PI3K/Akt/mTOR pathway by increasing the p-mTOR, p-Akt, and p-PI3K levels; however, the effects of LY294002 contrast with these results. Furthermore, there were no discernible variations in the total Akt, PI3K, or mTOR levels among the four groups.

**Figure 6:**
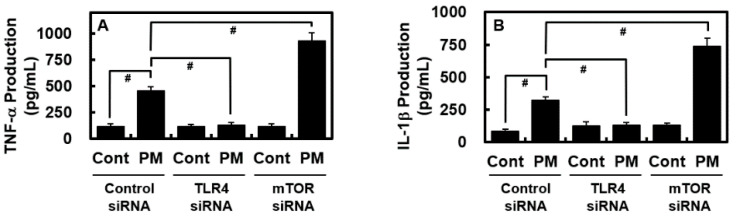
Effects of TLR4 and mTOR siRNAs on PM_2.5_-induced TNF-α and IL-1β production in purified MLMVECs. The isolated MLMVECs were transfected with TLR4 and mTOR siRNAs or control siRNA. Subsequently, these cells were exposed to PM_2.5_ (0.1 mg/mL) or the vehicle control for 6 h, and then TNF-α (**A**) and IL-1β (**B**) expression levels were examined using ELISA. Data were analyzed using ANOVA followed by Tukey’s post hoc test. Data are the means ± SD of three independent experiments (n = 3). ^#^ *p* < 0.01.

Genetic techniques were employed to block the expression of TLR4 and mTOR in isolated MLMVECs using TLR4 or mTOR siRNA to confirm that PM_2.5_-induced lung damage was dependent on the TLR4–mTOR signaling pathways. PM_2.5_ exposure drastically increased the expression levels of TNF-α and IL-1β in MLMVECs, and mTOR knockdown considerably increased TNF-α and IL-1β production (Figure 6). However, siRNA-mediated TLR4 knockdown substantially reduced PM_2.5_-induced TNF-α and IL-1β production (Figure 6).

## 3. Discussion

Lung toxicity caused by PM_2.5_ is closely related to the imbalance between autophagy and apoptosis-induced inflammation [15]. Thus, regulating the balance between autophagy and apoptosis may be a treatment strategy and a way to prevent lung diseases. CN exerts protective effects on the respiratory system similar to those of many other biologically active natural products. However, few studies to date have investigated the protective effect of CN against PM_2.5_-induced respiratory diseases. The present study was centered on the potential therapeutic use of CN for treating PM_2.5_-induced lung damage. Previous studies have demonstrated that PM enhances endothelium, epithelial, and macrophage inflammatory responses, leading to local lung inflammation [16,17,18]. Additionally, the overexpression of inflammatory mediators may cause systemic inflammation [16,17,18]. Thus, it is believed that inflammation is the primary physiological reaction to PM exposure.

We investigated the potential protective actions of CN against PM_2.5_-induced lung damage using mice. The findings suggested that CN exerted a protective effect against pulmonary edema by reducing the amount of total protein in the BALF, total inflammatory cell infiltration, lung W/D weight ratio, and hyperpermeability due to PM_2.5_ (Figure 1, Figure 2 and Figure 3). Polymorphonuclear leukocyte activation and oxidative stress have been assessed using MPO levels in terms of NO generation [19]. The TLR4–MyD88 pathway-mediated release of inflammatory mediators, such as IL-1β and TNF-α, activation of the inflammatory cascade, neutrophil migration to the alveoli, and lung damage constitute the most notable features of PM-induced pulmonary damage [20]. Our findings revealed that mice treated with CN for PM_2.5_-induced lung damage exhibited considerably lower levels of MPO, NO, and inflammatory cytokines, such as IL-1β and TNF-α than mice that were not treated (Figure 4). In our mouse model of PM_2.5_-induced lung injury, CN effectively suppressed both inflammatory cell infiltration into lung tissue and inflammatory cytokine production. The downregulation of TLR4 and MyD88 expression, the upregulation of mTOR phosphorylation, and the inhibition of autophagy are potential mechanisms underlying the suppression of PM_2.5_-induced lung injury via CN therapy.

In this study, mice were administered PM_2.5_ at a dose of 10 mg/kg body weight, which adequately induced lung damage and inflammation. Recent studies have reported that intratracheal PM_2.5_ instillation at 10 mg/kg body weight can cause cardiovascular dysfunction and respiratory diseases by inducing both local and systemic acute inflammation and/or simulating histological and functional changes in mouse lung tissue [21,22,23,24]. Intratracheal inhalation and instillation constitute the two main techniques of PM_2.5_ exposure in animal models. Mice, rats, and hamsters have been widely employed in intratracheal instillation procedures, which include injecting the required substance via a needle into the mouth and throat of the animals. We administered PM_2.5_ to the mice via intratracheal instillation. Intratracheal PM_2.5_ instillation reportedly causes lung damage, including pulmonary vascular hyperpermeability, alveolar epithelial dysfunction, and inflammatory responses [6,25]. Although this method has a number of disadvantages, such as being invasive and nonphysiological, bypassing the upper respiratory tract, and having confounding effects from the anesthesia and delivery vehicle [26], it needs to be performed only once, which improves accuracy and efficiency, thereby rendering it a simple and efficient technique for creating mouse models of lung damage [3].

During lysosome-dependent autophagy, damaged organelles, protein aggregates, and degraded cytoplasmic debris gather in autophagic vacuoles [10,11]. Reportedly, autophagy plays a role in the regulatory and developmental stages of lung damage and in healthy lung tissue, mTOR is activated and LC3 II, an autophagy-related protein, is downregulated; however, following lung damage, LC3 II is upregulated in human bronchial epithelial cells alongside mTOR downregulation [11]. Additionally, lipopolysaccharide (LPS)-induced p-mTOR is downregulated when TLR4 or MyD88 are knocked down, thereby revealing that the TLR4 signaling pathway activates mTOR and that LPS can suppress autophagy [27]. Because autophagy is controlled by a complex signaling network and TLR4 is a key autophagy sensor that has been heavily implicated in PM-induced immune responses, the involvement of a signaling network between TLR4 and autophagy is highly likely in PM-induced lung damage [28,29]. Both the mTOR–autophagy and TLR4–yD88 pathways have been hypothesized to influence lung injury as mTOR acts as a major suppressor of autophagy, which is involved in PM-induced lung inflammation [30].

The activation of the PI3K/Akt pathway can reportedly phosphorylate mTOR, a key regulator of autophagy [31]. By reducing autophagy and fostering lung recovery, p-mTOR can exert protective effects against lung injury [32,33]. Herein, CN considerably reduced the expression levels of LC3 II and Beclin 1 while substantially increasing those of p-mTOR, p-PI3K, and p-Akt. However, LY294002, a PI3K-specific inhibitor, strongly reversed the effects of CN, thereby indicating that CN inhibits excessive autophagy via PI3K/Akt/mTOR pathway activation. Additionally, our western blot assays revealed that CN decreased the expression levels of TLR4 and MyD88 (Figure 5B), suggesting that CN inhibits the PM_2.5_-induced upregulation of TLR4 and MyD88, thereby reducing the production of inflammatory cytokines, such as IL-1β and TNF-α, and oxidants, such as MPO and NO, in turn activating mTOR and autophagy in tissue cells.

PM is directly produced from various sources, including building sites, fires, smokestacks, and unpaved roadways. Thus, PM pollution can constitute a combination of hundreds of distinct substances in a wide range of sizes and morphologies. A drawback of this study is that it does not examine whether CN prevents the lung damage caused by various PM substituents.

In conclusion, we showed that CN reduced the lung W/D weight ratio, total protein levels, and lymphocyte counts, as well as inhibited inflammatory cell infiltration, inflammatory cytokine release, and PM_2.5_-induced hyperpermeability. Furthermore, by suppressing the TLR4 and autophagy pathways, CN aided in the recovery of tissue damage due to PM_2.5_-induced lung injury. Further examination regarding the effects of CN on the TLR4 and autophagy pathways and the PM_2.5_-induced inflammation will provide insight regarding its use in adverse health complications caused by PM_2.5_ exposure. As CN can be employed as a potentially effective therapeutic agent against PM_2.5_-induced lung damage, the findings of this study may help develop novel prevention and therapy techniques for PM-induced respiratory disorders.

## 4. Materials and Methods

### 4.1. Reagents

Diesel PM NIST 1650b (Sigma-Aldrich Inc., St. Louis, MO, USA) was mixed with saline in accordance with the manufacturer’s instructions and sonicated for 24 h to prevent the agglomeration of dispersed PM_2.5_ particles [34]. CN and dexamethasone (DEX, used as a positive control) were purchased from Sigma-Aldrich Inc. The negative control, mTOR, and TLR4 small interfering RNAs (siRNAs) were obtained from Santa Cruz Biotechnology (Santa Cruz, CA, USA).

### 4.2. Animal Care

Male Balb/c mice (7 weeks old, weighing ~27 g) were purchased from Orient Bio Co. (Sungnam, Republic of Korea) and employed in our investigation after acclimation for 12 days. The mice were handled according to the guidelines of Kyungpook National University regarding the care and use of laboratory animals (IRB #: KNU2019-103). The mice were categorized into eight groups (n = 10): the mock control group, the CN control group, the four PM_2.5_+CN groups (0.2, 0.4, 0.6, and 0.8 mg/kg mouse body weight), the DEX group (5 mg/kg mouse body weight), and the PM_2.5_ group (10 mg/kg mouse body weight in 50 μL of saline). An equivalent amount of phosphate-buffered saline (PBS) was administered to each of the control group mice. Subsequently, the mice were intratracheally administered with PM_2.5_ (10 mg/kg mouse body weight in 50 μL saline), and the CN and DEX groups received an injection into the tail vein as previously described [35]. The mice were euthanized 24 h following the injection, and their bronchoalveolar lavage fluid (BALF) and lung tissue were collected for future research purposes.

### 4.3. Wet/Dry Weight Ratio of the Lung Tissue

The right lung was weighed (wet weight) and then dried for 24 h at 120 °C in an oven before being weighed again (dry weight). The lung W/D weight ratio was calculated as a parameter of lung edema.

### 4.4. Culture of Mouse Lung Microvascular Endothelial Cell (MLMVECs) and siRNA Transfection

MLMVECs were treated as described previously [35,36]. Briefly, lung tissue was chopped and processed for 45–60 min at 37 °C with collagenase A (1 mg/mL). Anti-PECAM-1 monoclonal antibody (mAb) attached to magnetic beads (BD Pharmingen, San Diego, CA, USA) was used to separate the endothelial cells, which were then cultured in growth media for 2 days before purification. The cells were seeded on fibronectin-coated dishes and cultured in endothelial cell basal media supplemented with EGM-2 MV Bulletkit media (Lonza, Walkersville, MD, USA) to create a monolayer culture. The transfection of mouse mTOR, TLR4, or nonsense control siRNAs was performed as previously reported [35].

### 4.5. Hematoxylin and Eosin Staining

Formaldehyde solution (4%, Junsei, Tokyo, Japan) in PBS was used to fix the lung cells. Leftover blood was removed via three washings with PBS; the entire procedure took 20 h and was performed at 4 °C. Subsequently, the materials were embedded in paraffin blocks, dried, and sectioned into 4-m thick slices. Then, the slices were deparaffinized, rehydrated, and stained with hematoxylin and eosin (Sigma-Aldrich Inc.). The integrity of the pulmonary architecture and level of tissue edema were examined under a light microscope using a standard protocol by an observer who was blinded to the treatments [37].

### 4.6. Enzyme-Linked Immunosorbent Assay of p38, Mitogen-Activated Protein Kinase, Myeloperoxidase, Nitrous Oxide, Interleukon-1β, and TNF-α Phosphorylation

MLMVEC lysates were used to measure the amount of phosphorylated p38 mitogen-activated protein kinase (MAPK) using an enzyme-linked immunosorbent assay (ELISA) kit (Cell Signaling Technology, Danvers, MA, USA). The levels of myeloperoxidase (MPO), nitrous oxide (NO), IL-1β, IL-10, and TNF-α were measured in the BALF using ELISA kits according to the manufacturer’s instructions (R&D Systems, Minneapolis, MN, USA). ELISA plate readers (Tecan Austria GmbH, Grödig, Austria) were used for the ELISA readings.

### 4.7. Protein Concentration and Cell Count in the BALF

The BALF supernatant was centrifuged at 3000 rpm for 10 min at 4 °C, and the total protein concentration was measured using the QuantiProTM BCA Assay Kit (Sigma-Aldrich Inc.). The total protein concentration and cytokine levels were determined using the QuantiProTM BCA Assay Kit. A hematology analyzer was used to count the number of resuspended cells following resuspension in 50 μL PBS buffer.

### 4.8. Permeability Assays

The CN and DEX groups received injections via the tail vein at 30 min following the intratracheal dose of PM_2.5_ (10 mg/kg in 50 μL saline). A gas anesthetic machine was used to administer a 2% isoflurane-oxygen combination (Forane; JW Pharmaceutical, Seoul, Republic of Korea) for anesthesia (RC2 Rodent Circuit Controller; VetEquip, Pleasanton, CA, USA). The mice received face mask anesthesia prior to intravenous infusion of 1% Evans blue dye solution in regular saline. The mice were put to death by cervical dislocation after 6 h, and then the BALF was collected. ELISA plate readers were used to measure permeability data, as previously described [38,39,40].

### 4.9. Western Blot Analysis

The cells were first rinsed with ice-cold PBS and then treated with a RIPA lysis solution comprising 50 mM Tris-HCl (pH 7.5), 150 mM NaCl, 0.5% sodium dodecyl sulfate, 1% NP-40, 1% sodium deoxycholate, and protease inhibitors [41]. The blots were incubated with the primary antibodies after blocking with 5% bovine serum albumin for 2 h: anti-light chain (LC)3, anti-Beclin 1, anti-TLR4, anti-MyD88, anti-phosphorylated (p)mTOR, anti-Akt, anti-phosphorylated (p)PI3K, and anti-PI3K antibodies. (Cell Signaling Technology, Inc., Danvers, MA, USA). The membrane was then washed and incubated with secondary antibodies conjugated to horseradish peroxidase (Cell Signaling Technology, 1:10,000). ImageJ Gel Analysis tool (NIH, Bethesda, MD, USA) was used for concentration analyses.

### 4.10. Statistical Analysis

The data are presented as the means ± standard deviations (SD) for at least three separate trials for each experiment. Statistical relevance was estimated using one-way analysis of variance (ANOVA) and Tukey’s post hoc test, with a *p*-value of ≤0.05 considered statistically significant. SPSS (version 15.0, Chicago, IL, USA) for Windows was used for the statistical analyses.

## Data Availability

The data presented in this study are available upon reasonable request from the corresponding author.
